# Probiotic and technological characterization of selected *Lactobacillus* strains isolated from different egyptian cheeses

**DOI:** 10.1186/s12866-023-02890-1

**Published:** 2023-06-03

**Authors:** Mohsen Zommara, Shady El-Ghaish, Thomas Haertle, Jean-Marc Chobert, Mohamed Ghanimah

**Affiliations:** 1 Department of Dairy Science, Faculty of Agriculture, Kafrelsheikh University, Kafr El- Sheikh, 33516 Egypt; 2 UR 1268 Biopolymères Interactions Assemblages, Équipe Fonctions et Interactions des Protéines, INRA, Nantes Cedex 03, 44316 France

**Keywords:** *Lactobacillus*, Probiotic, Exopolysaccharide, Egyptian cheese

## Abstract

**Background:**

Fresh milk and natural environmental conditions are used to produce traditional cheeses. Such cheeses are produced by dozens of different types of microbes. Non-starter lactobacilli are the most responsible genus of lactic acid bacteria exhibiting key technological and health promoting traits. The purpose of this study is to isolate *Lactobacillus* bacteria from conventional Egyptian cheeses and analyse their probiotic potential and technological properties.

**Results:**

*Lactobacillus* isolates (33 isolates) were isolated from different Egyptian cheeses. Our results revealed that 18.18% of the isolates were fast-acidifying, 30.3% were medium-acidifying and 51.5% were slow-acidifying isolates. The results of autolytic activity showed that 24.3% of the isolates were good autolysis, 33.3% were fair autolysis, while 42.4% were poor autolysis. Fifteen isolates produced exopolysaccharides, while 9 isolates exhibited antimicrobial activities against *Lactobacillus bulgaricus* 340. All the isolates were resistant to pH 3 for 3 h except isolate No. 15 (MR4). The growth rate of the isolates ranged from 42.25 to 85.25% at 0.3% bile salts after 3 h of incubation. The surviving percentage of the *Lactobacillus* isolates decreased with increasing incubation time or the percentage of bile salts greater than 0.3%. All the isolates grew after incubation in artificial gastric and intestinal fluids. The auto-aggregation of 15 isolates ranged from 43.13 to 72.77%. *Lacticaseibacillus paracasei* BD3, *Lactiplantibacillus plantarum* BR4 and *Limosilactobacillus fermentum* MR2 were sensitive to the majority of the tested antibiotics and showed good bile salt hydrolase activity.

**Conclusion:**

*L. paracasei* BD3, *L. plantarum* BR4 and *L. fermentum* MR2 were isolated from Egyptian cheeses and showed probiotic and technological characterization, which are valuable for their practical application as starters, adjunct and protective cultures in cheese making.

## Introduction

Probiotics are live microorganisms that are ingested for their health advantages. Due to the ease of their digestion, they are frequently included in dietary supplements and functional foods [1]. The benefits of probiotics include improving the nutritional value of foods, preventing urinary tract and cardiovascular diseases, lowering cholesterol levels, reducing the risk of colon cancer, protecting against gut infections and diseases by boosting mucosal immunity and combating pathogenic bacteria [2]. Probiotics may have potential benefits for patients suffering from coronavirus infectious disease 2019 (COVID-19) due to their immunomodulatory properties [3, 4]. The selection requirements for probiotic lactic acid bacteria include safety, acid and bile resistance, activity/viability in the delivery vehicles, adherence to intestinal epithelial tissue, ability to colonize the gastrointestinal tract, secretion of antimicrobial substances, promotion of a host immune response without inflammation and ability to affect metabolic activities such as cholesterol assimilation and vitamin production [5]. The effectiveness of probiotics is strain specific, and the impact of one strain cannot be postulated for another strain unless clinical studies are confirmed [6].

The most common probiotics used in the food industry are bifidobacteria and genera of lactic acid bacteria (LAB), such as *Lactobacillus*, *Lactococcus, Streptococcus* and *Enterococcus* [7]. However, *Lactobacillus* strains are more resistant to oxygen than bifidobacteria strains [8].

The *Lactobacillus* genus is crucial for the manufacturing of fermented products [9]. *Lactobacillus* strains isolated from cheese and fermented milk products are generally recognized as safe. Recently, much attention has been focused on using them as probiotic and adjunct cultures in dairy products. *Lacticaseibacillus casei*, *Lacticaseibacillus paracasei*, *Lactiplantibacillus plantarum*, *Lacticaseibacillus rhamnosus* and *Lactobacillus acidophilus* have been isolated from cheese and fermented milk products as nonstarter lactic acid bacteria (NS-LAB) and have been defined as probiotics [10, 11].

Probiotic bacteria can produce extracellular carbohydrates known as exopolysaccharides (EPSs). The health interest in probiotic bacteria may be attributed to the production of EPSs, which can provide prevention against the harsh conditions of the digestive tract. Moreover, EPSs produced by probiotic strains may induce positive physiological reactions, such as lowering cholesterol and creating pathogenic biofilms through modulation of adhesion to epithelial cells [12]. The composition and biological functions of EPSs greatly depend on the microorganism type and environmental factors [13]. Consequently, it is crucial to select a suitable starter culture to optimise fermentation, organoleptic properties and safety aspects targeting modernistic products with a high added value. Additionally, since consumers request healthy products, manufacturers are continuously exploring the expansion of unfamiliar products, such as dairy products containing new probiotic strains, to appeal to health-conscious consumers [14].

Isolation and identification of LAB strains with potentially significant probiotic and technological characterization from several Egyptian cheeses may be useful for practical use as starter, adjunct, and protection cultures to improve traditional Egyptian dairy products.

Therefore, our objectives were to isolate and identify probiotic LAB from different traditional Egyptian cheeses based on their technological characteristics, which may play an important role in dairy manufacturing, health promoting and nutritional benefits.

## Materials and methods

### Materials

Tris-acetate-ethylene diamine tetra acetic acid (Tris-acetate-EDTA) was purchased from Thermo Fisher Scientific, USA. Pepsin, pancreatin, [sodium taurocholate, sodium cholate and sodium deoxycholate (sodium salt TDCA)], ethidium bromide, ox-bile salt and antibiotics (ampicillin, cefoxitin, doxycycline, streptomycin, neomycin, cephalexin, gentamycin, norfloxacin, ciprofloxacin and lincomycin) were obtained from Oxiod Ltd. (Altrincham, Cheshire, England WA14 2DT). MRS medium was obtained from BIOKAR Diagnostics, France. Agarose, agar, glycerol, di-potassium phosphate, sodium bicarbonate, hydrochloride acid, sodium hydroxide, hydrogen peroxide, potassium phosphate, toluene, sodium chloride, calcium chloride and potassium chloride were obtained from El Nasr Pharmaceutical Chemicals Company, Cairo, Egypt. Skim milk powder (Merck, Germany) was obtained from a local market. Defibrinated sheep blood was obtained from Thermo Scientific™ Oxoid.

### Sample collection

Samples of different Egyptian cheeses (45 samples) were collected during April and May from local markets of Lower Egypt cities, Tanta, El-Mehala and Basyoun, belong to Gharbia governorate located in northern Egypt. The collected cheese samples included Domiati cheese (rennet coagulated soft cheese), Ras cheese (ripened hard cheese manufactured from raw milk), and Kareish cheese (acid coagulated, low-fat soft cheese). Each sample was collected in sterile cups, transferred to the laboratory in an ice box and stored in a refrigerator.

### Isolation, purification and pre-identification of LAB

Isolation and pre- identification of LAB by morphological (rod- or sphere- shaped and colony characteristics) and physiological tests (Gram-positive and catalase-negative) were carried out [15–17]. The cultures were streaked onto MRS agar medium, and the purified strains were cultivated in reconstituted sterile skim milk (12.5%, w/v) with glycerol (30%, w/v) and stored at -20 °C.

### Technological characterization of selected *Lactobacillus* isolates

#### Acid production

The acidification rate was determined according to the method described by Ayad et al. [18].

### Autolytic activity

*Lactobacillus* isolates from exponentially growing cultures in MRS broth were harvested by centrifugation (5000 × *g* for 10 min at 4 °C). Pellets were washed and resuspended in 1 ml of potassium–buffered saline (PBS), pH 6.8. Lysis was monitored after 4 and 24 h of incubation at 37 °C by recording the reduction in optical density (OD) at 600 nm using a TOMOS UV-1800 spectrophotometer, Italy. The percentage of lysis was calculated as follows: [100 – (A1/A2)] x 100, where A1 (after incubation time) and A2 (before incubation time) are values of the OD_600_ [19].

### Exopolysaccharide (EPS) production

*Lactobacillus* isolates were streaked on MRS agar medium and incubated at 37 °C for 24 h. Cultures were tested for slime formation [20]. A metal loop was used to drag up the formed colonies, and the isolates were considered positive slime producers if the length of the slime was greater than 1.5 mm.

### Antimicrobial activity assay

The antimicrobial activity was determined by the well diffusion method [21] against *Lactobacillus bulgaricus* 340 [22, 23]. It was done using a 20 mL soft agar medium contains 0.8% agar and 100 µL of the indicator strain. The probable producer strains were centrifuged (8.000 rpm for 10 min at 4 °C) to obtain the cell-free supernatant. In order to eliminate the inhibitory impact of lactic acid on the test organisms, the pH of the supernatants was adjusted to 6.5 with 0.1 N NaOH and filtered through a 0.22 μm pore size filter. Agar was then divided into 9 mm-diameter wells, and 100 µL of the produced supernatant was added to each well. To allow for the radial diffusion of the substances present in the supernatant, plates were cooled at 4 ºC for 4 h prior to being incubated for 24 h at optimal temperature for the strain growth. Positive results were noted for a distinct clear zone of inhibition with a minimum diameter of 2 mm.

### Probiotic properties of *Lactobacillus* isolates

#### Resistance to low pH

Overnight cultures were propagated twice in MRS broth (0.1%, v/v) at 37 °C for 24 h to ensure that the cultures were in the logarithmic phase. Isolates were inoculated into MRS broth medium (10%, v/v) adjusted to pH 2.0, 3.0 and 4.0 using 1 M HCl solution and incubated at 37 °C. Bacterial growth was estimated by measuring the OD[11] at 600 nm after 3 and 24 h of incubation. The cultures were compared based on their growth development in each broth (pH 2.0, 3.0 and 4.0) and compared with the growth in the standard MRS broth (pH 6.0) (control). The differences (%) between the variations in OD at pH 6.0 and the variations in OD at pH 2.0, 3.0 and 4.0 generated an index of surviving isolates that was computed as follows: Surviving (%) = [(Δ OD pH_6_–OD pH_2,3 and 4_)/Δ OD pH_6_] x 100 [24].

An isolate survived if it showed a surviving percentage equal to or greater than 50%. All experiments were repeated two times in duplicate.

### Bile salt tolerance

Our isolates were inoculated into MRS broth (10%, v/v) containing ox-bile salt at a conc. of 0.3%, 0.5% and 1% (w/v). The OD was measured at 600 nm after 4 and 24 h of incubation at 37 °C and compared with the OD of culture without ox-bile salt (control) [11]. The results are expressed as the percentage of bile salt resistance as follows:

Survival (%) = (change in OD in MRS broth with ox-bile salt/change in OD in control MRS broth) × 100. Isolates showing a resistance percentage at a concentration of 0.3% of ox-bile salt higher than 50% were considered bile salt resistant isolates [24].

### Resistance to gastric acidity and bile salts

Pure cultures (10^8^ CFU/ml) of all isolates were exposed to artificial gastric fluid (0.72 g/L NaCl, 0.05 g/L KCl, 0.37 g/L NaHCO_3_, and 0.3 g/L pepsin) adjusted to pH 3.0 with 1 M HCl solution and to pH 7.0 with 1 M NaOH solution (control condition) for 0 and 3 h. After 3 h of incubation in artificial gastric fluid and in the control conditions, the bacteria were exposed to artificial intestinal fluid (0.3%, w/v, ox-bile salts and 0.1%, w/v, pancreatin, pH 8.0) for 0 to 5 h. Total viable counts were counted on MRS agar after a serial 10-fold dilution in PBS [25].

### Auto-aggregation

Isolates were grown in MRS broth for 24 h at 37 °C. The cells were harvested as previously described, washed, resuspended and diluted in sterile saline (0.85% NaCl) to OD_600 nm_ = 0.3. One millilitre of the cell suspension was transferred to a sterile plastic cuvette, and the OD_600 nm_ was recorded after 60 min. Auto-aggregation was computed using the following equation: %Auto-aggregation = [(OD_0_ - OD_60_)/OD_0_] × 100.

OD_0_ is the initial OD value, and OD_60_ is the OD value after 60 min. For determination of OD_60_, the cultures were centrifuged at 300 × *g* for 2 min at 20 °C [26].

### Hydrophobicity

The hydrophobicity assay was carried out to test the ability of the isolates to adhere to the selected hydrocarbons [27] with minor modifications. Isolates were grown in MRS broth at 37 °C for 24 h. Cells were harvested (8000 × g for 6 min at 4 °C), washed twice with PBS and resuspended in the same solution, and the OD_600 nm_ was measured. A total of 1.5 ml of cell suspension was mixed with 1.5 ml toluene and vortexed for 2 min. The aqueous and organic phases were allowed to separate for 30 min at room temperature. The aqueous phase was removed, and the OD_600 nm_ was recorded. The experiment was repeated, and the average OD value was determined. The percentage hydrophobicity was computed as follows:

% Hydrophobicity = [(OD _600_ reading 1 - OD_600_ reading 2)/OD _600_ reading 1] ×100.

A higher hydrophobicity percentage indicates a higher colonization or adherence potential of bacteria in the gut [6].

### Bile salt hydrolase (BSH)

Isolates were tested for BSH activity by streaking cultures grown in MRS broth onto BSH screening medium, which consisted of MRS agar containing sodium salt of TDCA (taurodeoxycholic acid) at a conc. of 0.5% (w/v) and CaCl_2_ (0.37 g/L). Plates were incubated anaerobically at 37 °C. The bile salt hydrolase activity was semiquantified by the precipitation zones. The assay was performed in duplicate [27].

### Haemolytic activity

The isolates were streaked onto MRS agar fortified with defibrinated sheep blood (5%) to test for haemolysis [28]. Each bacterial suspension was streaked onto plates. After incubation (24 h/37°C), the plates were screened for signs of α-haemolysis (a green-hued zone around colonies), β-haemolysis (clear zones around colonies) or γ-haemolysis (no halo around colonies).

### Antibiotic susceptibility test

It was determined by using the disc diffusion method. This method was used to screen for the antibiotic susceptibility of isolates with 10 discs: AML_10_-ampicillin (10 µg), FOX_10_-cefoxitin (10 µg), DO_30_-doxycycline (30 µg), S10-streptomycin (10 µg), N_30_-neomycin (30 µg), CL30-cephalexin (30 µg), CN_10_-gentamycin (10 µg), NOR_10_-norfloxacin (10 µg), CIP5-ciprofloxacin (5 µg), and L_10_-lincomycin (10 µg) [29].

### Molecular identification of selected LAB isolates

The isolate DNA was extracted [30] and used as a template for 16 S rRNA gene amplification. In the reaction, the universal primers fD1 (5’-AGAGTTTGATCCTGGCTCAG-3’) and rD1 (5’-TAAGGAGGTGATCCAGGC-3’) were used [31]. DNA amplifications were carried out in a DNA thermal cycler model (Techno, Barloworld Scientific, Cambridge, UK). A final extension was performed for 5 min at 72 °C. Amplicons were analysed on agarose gel (1%, w/v) with ethidium bromide (0.5 mg/ml) in 0.5x TAE (Tris acetate-EDTA) buffer for 30 min at 100 V and visualized with UV trans-illumination. DNA sequencing was carried out by MilleGen sequencing services (Labège, France).

### Statistical analysis

Data are expressed as averages ± standard error with Statistical Package for Social Studies software (SPSS, version 16).

## Results and discussion

### Isolation and pre-identification of lactic acid bacteria (LAB)

Two hundred fifty-six isolates were isolated from different Egyptian cheeses (Table 1). LAB are catalase-negative and Gram-positive heterogeneous group of bacteria that play a significant role in a variety of fermentation processes. The results in Table 1 show that the ratio of non-LAB to LAB was 40.2–59.8%. Beukes et al. [32] reported that LAB were dominant in traditional fermented milk in South Africa. The isolated LAB were classified into 33 and 120 rod and coccus isolates, respectively, revealing the preponderance of coccus over *Lactobacillus* (Table 1), which is consistent with prior findings [18, 33, 34].

**Table 1:** Pre-identification of LAB isolates in cheese samples collected from Gharbia Governorate

City	Cheese type	Number of samples	Bacteria isolates counts		LAB	
			Cocci	Rods	Cocci	Rods
Tanta	Domiati cheese	5	19	8	8	4
	Kareish cheese	5	16	6	9	3
	Ras cheese	5	11	5	8	1
El-Mehala	Domiati cheese	5	36	5	22	2
	Kareish cheese	5	19	8	9	5
	Ras cheese	5	18	5	10	3
Basyoun	Domiati cheese	5	33	8	20	6
	Kareish cheese	5	32	9	26	6
	Ras cheese	5	14	4	8	3
Total		45	198	58	120	33
Total isolates			256		153	
Ratio of non-LAB : LAB			40.23%		59.77%	
Ratio of cocci : rods			77.3%	22.7%	78.4%	21.6%

The ratio of non-LAB: LAB = (100 – ratio of LAB) (number of LAB/number of all isolates) x100

The ratio of cocci:rods = (number of cocci/number of all isolates) x 100: (100 – ratio of cocci)

### Technological characterization of selected *Lactobacillus* isolates

#### Acid production

Table 2 illustrates that 18.2% of the tested *Lactobacillus* isolates (6 isolates) were fast-acidifying, 30.3% (10 isolates) were medium-acidifying and 51.5% (17 isolates) were slow-acidifying. Our findings are consistent with those of Ayad et al. [18], who indicated that the majority of *Lactobacillus* isolates were slow acidifiers in milk (58%). Fermentation of lactose and production of lactic acid by selected LAB starters is an essential process during cheese manufacture and in cheese ripening. A rapid reduction in pH is regarded crucial because it is required for coagulation, curd/cheese hardness and inhibition of unwanted microorganisms[35]. Nonstarter or secondary LAB cultures play a significant role during cheese ripening, but they do not contribute to acid production [35]. Consequently, fast-acidifying isolates are good candidates for primary starter organisms in dairy fermentation processes, whereas slow-acidifying strains can be employed as adjunct cultures [18]. Meng et al. [36] reported that most of the lactobacilli strains isolated from semihard goat cheeses exhibited low acidification activity. In addition, they found that *L. paracasei* and *L. rhamnosus* strains displayed slow acidification activity [36].

**Table 2:** Technological properties of *Lactobacillus* isolates

	*Acid production			†Autolytic activity			‡Exopolysaccharide (EPS) production		**Antimicrobial activity			
	Fast	Medium	Slow	Good	Fair	Poor	Yes	No	High	Medium	Low	Non
Number of *Lactobacillus* isolates	6	10	17	8	11	14	15	18	1	3	5	24
Percentage of *Lactobacillus* isolates	18.2	30.3	51.5	24.3	33.3	42.4	45.4	54.6	3.0	9.1	15.2	72.7
Total Isolates	33			33			33		33			

Fast, medium and slow; when a pH of 0.4 U was achieved after 3, 3–5 and > 5 h, respectively [18]

†Good, 35–66%; fair, 24–34%; poor, 0–23% [18]

‡The percentage was calculated as (number of isolates/number of total isolates) x100

**High activity (inhibition zone > 6 mm), medium activity (inhibition zone = 3–6 mm), low activity (inhibition zone < 3 mm) and no activity (inhibition zone = 0 mm)

### Autolytic activity

The autolytic activity of bacteria is of great importance in cheese ripening because it leads to the release of intracellular proteolytic enzymes, particularly peptidases. These enzymes hydrolyse peptides in cheese and produce amino acids, which are in turn responsible for the emergence of flavouring elements [35]. The autolytic activities of the collected LAB isolates were found to be different depending on the isolate and were evaluated as poor, fair, or good. The data in Table 2 show that the number of good *Lactobacillus* isolates was 8 (24.3%), fair autolysis isolates was 11 (33.3%) and poor autolysis isolates was 14 (42.4%). These results agree with those obtained by Ayad et al. [18]. Meng et al. [36] found that 56% of lactobacilli isolates were rated good autolysis, 32% were rated fair autolysis and the remainder were rated poor autolysis. The discrepancies in the autolytic percentages of isolates reflect the vast variety of strains. The inclusion of adjunct cultures, mostly *Lactobacillus* spp., is one of the best ways to hasten cheese ripening, and the choice of these cultures should be based on their enzyme profiles and autolytic capabilities [37].

### Exopolysaccharide (EPS) production

Many LAB strains generate EPSs, which can be tightly bound to the bacterial cell, loosely bound, or discharged as slime [38]. Slime-forming LAB have been widely employed as natural biothickeners in the dairy sector. In addition to the textural features conferred by acid generation, they contribute to thickening fermented products and give them key textural properties [18]. The results revealed the presence of 15 (45.4%) EPS-producing *Lactobacillus* isolates in our culture collection (Table 2) of 33 tested *Lactobacillus* isolates. Our findings are consistent with those of Ayad et al. [18], who found that some *Lactobacillus* isolates produce EPSs. The results of Tarique et al. [11] showed that all the selected LAB isolates from Labneh (except 2 isolates) were found to produce EPS. Meng et al. [36] found that 11 *L. paracasei* and 6 *L. rhamnosus* strains isolated from semihard goat cheeses produced EPS. EPS-producing cultures are used to enhance the textural, rheological, and organoleptic properties of low-fat cheeses and fermented milks [38].

### Antimicrobial activity assay

Nine isolates (27.27%) exhibited antibacterial action against *L. bulgaricus* 340. The antimicrobial isolates were divided into three categories according to their antimicrobial activity: high (1 isolate), medium (3 isolates) and low (5 isolates) (Table 2). Our results are higher (27.3%) than that reported by Ayad et al. [18], who found that 21% of 269 *Lactobacillus* isolates appeared to have antimicrobial activity. These differences may be attributed to the isolates. Atanassova et al. [22] found that *L. paracasei* subsp. *paracasei* strain M3 can produce an active proteinaceous substance which exhibited bactericidal and fungistatic activities. Additionally, this material displayed antimicrobial activity against *L. delbrueckii* species.Bacteriocins active against LAB from the same or nearly similar species can be produced by LAB. Bacteriocins, hydrogen peroxide, organic acids, and diacetyl are antimicrobial compounds produced by LAB that are hostile to spoilage and pathogenic organisms [39]. To increase cheese safety and quality, bacteriocin-producing strains have been utilised as starter or adjunct cultures [40].

### Probiotic strains

The EPS-producing *Lactobacillus* isolates were tested for bile tolerance, resistance to low pH, gastric acidity and bile salts, haemolytic activity, bile salt hydrolase, auto-aggregation, hydrophobicity, and antibiotic susceptibility.

### Resistance to low pH

To assess the prospective use of LAB as efficient probiotics, it is widely assumed that their capacity to withstand the effects of acids must be determined [41]. Food typically remains in the stomach for 3 h, and this time constraint was considered. Therefore, strains showing more than 50% resistance to pH 3 for 3 h were considered resistant to low pH [42]. Table 3 shows the survival rates at pH 2, 3, and 4. The obtained data indicate that the percentages of surviving *Lactobacillus* isolates varied from 48.09 to 72.02% at pH 3 for 3 h. All the isolates were resistant at pH 3 for 3 h except isolate No. 15 (MR4). All isolates were less resistant < 50% after 24 h of incubation. Our findings are similar to those of François et al. [24], who found that most *Lactobacillus* strains (34 isolates) were resistant to pH 3 after 6 h of exposure, while the survival reduced with prolong incubation time. In addition, Handa and Sharma [42] studied the potential acid tolerance of *L. plantarum* F22 and demonstrated its resistance to pH 3. Guan et al. [43] determined the effect of an acidic environment on the viability of *L. plantarum* HLX37 and reported that the strain survived well under acidic conditions (pH ≥ 2.5). Prabhurajeshwar and Chandrakanth [44] reported that *Lactobacillus* isolates are adapted to grow in both acidic and natural conditions.

**Table 3:** Surviving percentages of *Lactobacillus* isolates in MRS broth incubated at 37 °C for 3 and 24 h at pH 2, 3 and 4

Isolates	Code	Surviving percentage (%)					
		pH 2		pH 3		pH 4	
		3 h	24 h	3 h	24 h	3 h	24 h
1	TK1	46.04 ± 5.84	28.76 ± 3.66	52.28 ± 0.28	34.64 ± 4.43	60.52 ± 1.48	46.45 ± 0.88
2	TK3	45.41 ± 1.10	24.23 ± 4.02	58.54 ± 2.47	37.02 ± 2.08	59.89 ± 5.11	57.69 ± 2.52
3	TD2	43.82 ± 0.70	31.70 ± 5.48	51.89 ± 1.59	47.77 ± 2.05	53.93 ± 5.07	51.40 ± 1.20
4	TR1	48.91 ± 6.36	29.82 ± 3.65	68.73 ± 5.28	42.61 ± 2.09	69.59 ± 4.42	47.47 ± 5.65
5	TR3	56.99 ± 8.31	27.96 ± 0.18	61.22 ± 8.78	44.30 ± 8.93	66.50 ± 6.50	44.90 ± 7.31
6	BK2	54.04 ± 0.32	19.32 ± 3.99	58.10 ± 2.10	31.58 ± 7.35	57.57 ± 6.44	48.53 ± 2.59
7	BD2	48.98 ± 5.78	22.24 ± 4.98	56.18 ± 6.18	30.43 ± 4.17	59.04 ± 1.04	48.61 ± 7.50
8	BD3	51.88 ± 5.73	24.63 ± 3.53	72.02 ± 2.13	36.51 ± 0.35	60.66 ± 5.35	49.91 ± 1.21
9	BR1	55.31 ± 4.05	29.02 ± 1.24	53.24 ± 5.00	37.28 ± 0.98	69.49 ± 2.51	45.83 ± 4.53
10	BR3	49.17 ± 3.13	32.08 ± 8.04	57.12 ± 0.79	45.25 ± 0.65	59.90 ± 8.10	44.78 ± 2.43
11	BR4	43.84 ± 5.67	32.11 ± 2.89	68.17 ± 3.84	38.81 ± 1.40	66.59 ± 9.42	55.95 ± 9.28
12	MK1	50.85 ± 0.40	28.76 ± 1.25	58.72 ± 9.69	36.79 ± 1.47	48.31 ± 5.69	39.72 ± 0.31
13	MK3	51.25 ± 1.02	24.70 ± 3.45	51.67 ± 3.51	35.89 ± 3.67	57.85 ± 3.15	46.99 ± 1.25
14	MR2	49.87 ± 2.44	27.64 ± 3.43	64.34 ± 5.97	33.59 ± 4.38	67.29 ± 3.72	46.05 ± 1.92
15	MR4	41.82 ± 5.56	28.57 ± 0.69	48.09 ± 4.93	33.86 ± 1.15	70.69 ± 3.32	60.07 ± 0.16

T = Tanta; B = Basyoun; M = El-Mehala; K = Kareish cheese; D = Domiati cheese; R = Ras cheese

### Bile tolerance

Bacteria used as probiotics are typically fed to animals and hence must be able to withstand harsh circumstances in the digestive system, including bile discharges. Therefore, bile tolerance was thought to be necessary for bacterial colonisation and metabolic activity in the host’s gut [44]. It is estimated that the average intestinal bile concentration is 0.3% (w/v). As a result, while evaluating the likelihood of utilising LAB as efficient probiotics, it is commonly assumed that their capacity to withstand the effects of bile salts is required [41]. In this study, the ability of the *Lactobacillus* isolates to tolerate bile was investigated. After 3 h of incubation, the growth rate of the *Lactobacillus* isolates ranged from 42.25 to 85.25% at 0.3% bile salts (Table 4). The data in Table 4 also show an inverse relationship between the *Lactobacillus* isolate survival percentage and incubation time or an increase in the percentage of bile salts by more than 0.3%. Our results are in accordance with those obtained by François et al. [24], who found that all *Lactobacillus* isolates showed ≥ 50% survival percentages at 0.2 and 0.4% (w/v) bile salts. Additionally, Handa and Sharma [42] found that *L. plantarum* F22 was resistant to 0.3% bile concentration. Guan et al. [43] reported that *L. plantarum* HLX37 tolerates high concentrations of bile salts. The acid and bile tolerances varied between isolates within the same species, which has been observed previously for both *Lactobacillus* isolates. The resistance of probiotics to different bile salts varies depending on the strain [11]. The presence of polysaccharides on the outer cell membrane could be responsible for the resistance of bacteria to bile salts [45].

**Table 4:** Surviving percentage of *Lactobacillus* isolates in MRS broth supplemented with 0.3, 0.5 or 1.0% Ox-bile after 4 and 24 h at 37 °C

Isolates	Code	Ox-bile, %					
		0.3%		0.5%		1.0%	
		3 h	24 h	3 h	24 h	3 h	24 h
1	TK1	46.37 ± 4.36	51.69 ± 6.19	43.22 ± 4.46	50.03 ± 1.42	25.86 ± 3.52	30.79 ± 4.09
2	TK3	78.40 ± 4.17	51.13 ± 4.22	52.50 ± 9.90	55.44 ± 6.74	37.41 ± 5.80	33.08 ± 3.28
3	TD2	59.69 ± 6.37	48.11 ± 5.90	50.36 ± 4.03	55.53 ± 5.09	26.85 ± 5.63	35.08 ± 3.48
4	TR1	52.47 ± 9.74	49.49 ± 3.22	44.38 ± 5.29	46.65 ± 1.84	34.32 ± 0.87	32.73 ± 5.10
5	TR3	49.29 ± 3.65	46.30 ± 6.17	38.88 ± 5.24	50.73 ± 4.50	53.39 ± 5.82	27.39 ± 3.10
6	BK2	59.18 ± 3.95	54.93 ± 6.30	44.99 ± 6.72	50.40 ± 5.21	26.61 ± 4.44	37.79 ± 2.59
7	BD2	61.46 ± 7.76	50.54 ± 8.42	44.49 ± 7.45	44.45 ± 0.89	26.02 ± 2.51	41.01 ± 1.76
8	BD3	58.44 ± 1.85	77.07 ± 5.49	43.58 ± 6.78	48.81 ± 7.49	34.74 ± 7.17	39.57 ± 1.95
9	BR1	66.31 ± 6.12	52.64 ± 3.59	41.89 ± 2.46	58.16 ± 4.79	24.57 ± 6.37	39.51 ± 3.25
10	BR3	85.25 ± 5.69	60.16 ± 7.53	46.40 ± 4.23	59.03 ± 5.91	39.16 ± 1.64	41.54 ± 0.82
11	BR4	85.08 ± 7.85	83.73 ± 5.53	64.83 ± 6.46	45.19 ± 4.38	61.27 ± 3 0.91	44.87 ± 3.13
12	MK1	71.08 ± 6.70	45.48 ± 6.21	66.19 ± 4.63	52.34 ± 5.92	30.31 ± 3.34	34.46 ± 2.17
13	MK3	42.25 ± 0.72	55.49 ± 7.07	53.59 ± 6.23	56.41 ± 5.91	27.91 ± 4.44	35.93 ± 1.70
14	MR2	78.50 ± 5.11	49.30 ± 5.91	46.26 ± 6.11	49.38 ± 4.98	26.20 ± 2.64	28.39 ± 3.71
15	MR4	43.24 ± 7.13	44.55 ± 3.82	58.48 ± 2.18	47.16 ± 4.08	27.02 ± 1.49	31.57 ± 2.21

T = Tanta; B = Basyoun; M = El-Mehala; K = Kareish cheese; D = Domiati cheese; R = Ras cheese

### Resistance to artificial gastric and intestinal fluids

A probiotic must survive gastrointestinal transit before it may be useful. pH 2 was the essential limit for probiotic strain viability in acidic conditions, which efficiently inhibited the survival of strains [46]. Therefore, strains with high survival rates were selected for their ability to cross the human intestinal barrier. The data in Table 5 show that all isolates grew after incubation in artificial gastric and intestinal fluids, indicating that the enzymatic activity of pepsin had no effect on the 15 strains. After 5 h of exposure, all strains showed similar levels of pancreatin resistance, with viability dropping to fewer than 2.5 log CFU/ml. (Table 5). These findings were consistent with those of Tarique et al. [11] and Jamaly et al. [47].

**Table 5:** Count of *Lactobacillus* spp. in the artificial gastric and intestinal fluids

Isolates	Code	log CFU/ml			
		3 h incubation in gastric fluid		5 h incubation in intestinal fluid	
		pH 7 Initial count	pH 3 Final count	0 h Initial count	5 h Final count
1	TK1	6.62 ± 0.42	6.73 ± 0.53	6.22 ± 0.32	5.43 ± 0.39
2	TK3	6.34 ± 0.74	6.23 ± 0.93	5.67 ± 0.57	5.46 ± 0.38
3	TD2	6.83 ± 0.13	7.03 ± 0.23	6.40 ± 0.10	5.71 ± 0.76
4	TR1	6.59 ± 0.59	6.58 ± 0.58	6.03 ± 0.23	4.89 ± 0.29
5	TR3	6.30 ± 0.60	6.18 ± 0.51	5.62 ± 0.92	4.40 ± 0.50
6	BK2	6.40 ± 0.90	6.58 ± 0.58	5.99 ± 0.29	4.80 ± 0.50
7	BD2	6.10 ± 0.50	6.17 ± 0.51	5.52 ± 0.92	4.94 ± 0.17
8	BD3	7.02 ± 0.02	7.01 ± 0.04	6.28 ± 0.32	4.67 ± 0.37
9	BR1	6.21 ± 0.91	6.23 ± 0.93	5.52 ± 0.72	5.06 ± 0.06
10	BR3	7.02 ± 0.02	6.92 ± 0.09	5.81 ± 0.01	4.82 ± 0.22
11	BR4	7.01 ± 0.11	7.08 ± 0.18	6.48 ± 0.02	4.21 ± 0.91
12	MK1	6.39 ± 0.69	6.42 ± 0.72	5.62 ± 0.62	5.11 ± 0.03
13	MK3	6.70 ± 0.30	6.82 ± 0.42	6.18 ± 0.38	4.45 ± 0.55
14	MR2	7.23 ± 0.07	7.27 ± 0.03	6.62 ± 0.09	4.30 ± 0.86
15	MR4	6.15 ± 0.75	6.33 ± 0.93	5.59 ± 0.89	4.84 ± 0.06

T = Tanta; B = Basyoun; M = El-Mehala; K = Kareish cheese; D = Domiati cheese; R = Ras cheese

### Auto-aggregation

Aggregation between bacterial cells of the same strain (auto-aggregation) is significant and is one of the important variables in determining the lactic acid bacterial strain’s capacity to attach to the mouth cavity and gastrointestinal tract [48]. Aggregation is a critical component in biofilm development [49]. The sedimentation rate of the *Lactobacillus* isolates was determined after 60 min in the current investigation. The auto-aggregation of several *Lactobacillus* isolates ranged from 43.13 to 72.77%, according to the results in Table 6. These findings show that *Lactobacillus* isolate No. 8 (BD3) has a considerable adhesive capacity, which is favorable for robust adhesion in the digestive tract. The observed auto-aggregation is related to the cell surface component that was not lost after washing and suspending the cells in PBS. Our findings are consistent with those of other studies [42–44, 49].

**Table 6:** Auto-aggregation and hydrophobicity and bile salt hydrolase (BSH) and hemolysis activities of different isolates

Isolates	Code	Auto aggregation	Hydrophobicity %	BSH activity	Hemolysis
1	TK1	45.87 ± 6.63	34.60 ± 4.16	-	α
2	TK3	48.13 ± 7.22	49.90 ± 6.10	-	γ
3	TD2	43.13 ± 8.32	49.20 ± 5.91	-	γ
4	TR1	44.90 ± 7.73	42.43 ± 3.70	-	γ
5	TR3	56.33 ± 3.89	42.00 ± 4.76	-	γ
6	BK2	53.67 ± 2.96	37.53 ± 2.83	+	γ
7	BD2	49.93 ± 4.74	35.27 ± 3.58	+	γ
8	BD3	72.77 ± 6.79	83.00 ± 3.21	+	γ
9	BR1	57.40 ± 2.25	74.13 ± 3.79	-	α
10	BR3	53.07 ± 3.07	31.03 ± 4.14	+	γ
11	BR4	63.23 ± 7.02	66.23 ± 5.81	+	γ
12	MK1	65.37 ± 3.70	74.90 ± 4.54	-	γ
13	MK3	68.10 ± 4.28	73.77 ± 3.87	+	β
14	MR2	65.40 ± 3.22	61.90 ± 5.80	+	γ
15	MR4	66.07 ± 3.94	63.43 ± 2.91	-	α

T = Tanta; B = Basyoun; M = El–Mehala; K = Kareish cheese; D = Domiati cheese; R = Ras cheese

### Hydrophobicity

A nonspecific interaction between microbial cells and the host is defined as cell surface hydrophobicity. High hydrophobicity bacterial cells typically interact strongly with mucosal cells [49]. Bacterial cell adhesion is highly linked to cell surface features. In vitro adhesion of probiotic isolates to epithelia was investigated by measuring cell surface hydrophobicity towards toluene. In all *Lactobacillus* isolates, the estimated hydrophobicity varied from 31.03 to 83% (Table 6). Isolates 8 (BD3) and 9 (BR1) were very hydrophobic (Table 6). Other studies have shown similar findings [44, 47, 49]. The discrepancies in hydrophobicity might be attributed to changes in the amount of expression of surface proteins of cells among isolates as well as environmental factors that may influence expression [6].

### Bile salt hydrolase

Bile salt hydrolase (BSH) is an essential element because it may help to maintain the balance of gut bacteria while lowering blood cholesterol [49]. Isolates No. 6 (BK2), 7 (BD2), 8 (BD3), 10 (BR3), 11 (BR4), 13 (MK3) and 14 (MR2) had good BSH activity, which suggests they are able to reduce cholesterol. Similar results were obtained by Tarique et al. [11], Agaliya and Jeevaratnam [49].

### Haemolytic activity

For safety reasons, it is critical to guarantee the lack of haemolytic activity in the *Lactobacillus* isolates under investigation, allowing for their acceptable and secure use as starter or adjunct cultures in food systems. Haemolysis is a virulence factor frequently associated with pathogenic microorganisms. The data in Table 6 show that *Lactobacillus* isolates No. 2 (TK3), 3 (TD2), 4 (TR1), 5 (TR3), 6 (BK2), 7 (BD2), 8 (BD3), 10 (BR3), 11 (BR4), 12 (MK1) and 14 (MR2) had γ-haemolytic activity, whereas No. 1 (TK1), 9 (BR1) and 15 (MR4) had α-haemolytic activity, and isolate No. 13 (MK3) had β-haemolytic activity. Previous studies have found that most lactobacilli species do not possess haemolytic activities, with the exception of a few strains that exhibited α-haemolytic activity [47, 49].

### Antibiotic susceptibility test

One of the essential requirements for safety and approval for use of the bacteria as starter or adjunct cultures in food systems is antibiotic susceptibility. Due to the potential for passing antibiotic resistant genes to intestinal pathogens, safety concerns surrounding the use of probiotics harbouring antibiotic-resistant strains have grown. However, because the antibiotic resistance found in *Lactobacillus* strains is chromosomally encoded and hence non-transmissible, earlier studies have suggested that it is intrinsic or natural resistance. The *Lactobacillus* genus is thought to be intrinsically resistant to aminoglycoside antibiotics such as gentamicin, streptomycin, and kanamycin, and this resistance is due to the lack of cytochrome-mediated electron transport, which facilitates drug absorption [50]. The antibiotic sensitivity test clarified that isolates No. 8 (BD3), 11 (BR4) and 14 (MR2) were sensitive to the majority of tested antibiotics, but the other isolates were resistant to all (7 isolates) or most (4 isolates) of the studied antibiotics (Table 7). As shown in Table 7, isolate No. 8 was resistant to doxycycline and lincomycin; isolate No. 11 was resistant to cefoxitin, norfloxacin and lincomycin; and isolate No. 14 was resistant to neomycin and lincomycin. The probable reason for antibiotic resistance among strains may be due to horizontal transmission of antibiotic-resistant genes [6]. Our results were in accordance with some authors and in disagreement with others. François et al. [24] reported that all tested *Lactobacillus* isolates were sensitive to ampicillin. Handa and Sharma [42] found that *L. plantarum* F22 was sensitive to gentamycin, ampicillin and ciprofloxacin. Guan et al. [43] revealed that *L. plantarum* isolates were sensitive to cephalexin and gentamycin but resistant to streptomycin. Prabhurajeshwar and Chandrakanth [44] demonstrated that most *Lactobacillus* isolates were sensitive to gentamycin, ampicillin and ciprofloxacin. Jamaly et al. [47] found that *Lactobacillus* isolates were sensitive to gentamycin and ampicillin. Agaliya and Jeevaratnam [49] found that *L. plantarum* isolates were sensitive to streptomycin, ampicillin and gentamycin, but resistant to gentamycin, ciprofloxacin and norfloxacin. Puniya et al. [51] reported that *Lactobacillus* isolates were sensitive to streptomycin but resistant to gentamycin.

**Table 7:** Susceptibility of *Lactobacillus* isolates against some antibiotics measured by agar disc diffusion

Isolates	Code	Disc antibiotic (µg)									
		AML_10_	FOX_10_	DO_30_	S_10_	N_30_	CL_30_	CN_10_	NOR_10_	CIP_5_	L_10_
1	TK1	R	R	R	R	R	R	R	R	R	R
2	TK3	S	R	R	R	R	R	R	R	R	R
3	TD2	R	R	R	R	R	S	R	R	R	R
4	TR1	R	R	R	R	R	R	R	R	R	R
5	TR3	R	S	R	R	R	R	R	R	S	R
6	BK2	R	R	R	R	R	R	R	R	R	R
7	BD2	R	R	R	R	R	R	R	R	R	R
8	BD3	S	S	R	S	S	S	S	S	S	R
9	BR1	R	R	R	S	R	R	R	R	R	R
10	BR3	R	R	R	R	R	R	R	R	R	R
11	BR4	S	R	S	S	S	S	S	R	S	R
12	MK1	R	S	R	R	R	R	R	S	R	R
13	MK3	R	R	R	R	R	R	R	R	R	R
14	MR2	S	S	S	S	R	S	S	S	S	R
15	MR4	R	R	R	R	R	R	R	R	R	R

R: resistant; S: sensitive; AML_10_-ampicillin; FOX_10_- cefoxitin; DO_30_- doxycycline; S10- streptomycin; N_30_ – neomycin; CL30 – cephalexin; CN_10_ – gentamycin; NOR_10_ – norfloxacin; CIP5 – ciprofloxacin; L_10_ – lincomycin

T = Tanta; B = Basyoun; M = El-Mehala; K = Kareish cheese; D = Domiati cheese; R = Ras cheese

### Molecular identification of selected LAB isolates

In light of their probiotic and technical attributes, the ideal *Lactobacillus* isolates were selected for identification by 16 S rDNA. Isolate No. 8 (BD3) is *L. paracasei* BD3, isolate No. 11 (BR4) is *L. plantarum* BR4 and isolate No. 14 (MR2) is *L. fermentum* MR2.

## Conclusions

In conclusion, a number of LAB were found in numerous cheeses from Lower Egypt. They exhibit potentially technologically interesting traits (probiotic and technical characterisation) that may be used practically as starters, adjuncts and protective cultures. To improve Egyptian fermented dairy products, subsequent research will assess how the chosen strains behave in small-scale cheese production, fermented milk production, and mixed cultures.

## Data Availability

The datasets generated and/or analyzed during this study are available from the corresponding author upon reasonable request.
